# Increased Risk for Invasive Group A *Streptococcus* Disease for Household Contacts of Scarlet Fever Cases, England, 2011–2016

**DOI:** 10.3201/eid2503.181518

**Published:** 2019-03

**Authors:** Vicky Watts, Sooria Balasegaram, Colin S. Brown, Suzanna Mathew, Rachel Mearkle, Derren Ready, Vanessa Saliba, Theresa Lamagni

**Affiliations:** Public Health England, Liverpool, UK (V. Watts);; Public Health England, London, UK (S. Balasegaram, C.S. Brown, D. Reddy, V. Saliba, T. Lamagni);; Public Health England, Leeds, UK (S. Mathew);; Public Health England, Chilton, UK (R. Mearkle)

**Keywords:** invasive group A Streptococcus disease, iGAS disease, scarlet fever, streptococcal infections, Streptococcus pyogenes, bacteria, streptococci, respiratory infections, household contacts, incidence, risk, England

## Abstract

The incidence of scarlet fever in England and Wales is at its highest in 50 years. We estimated secondary household risk for invasive group A *Streptococcus* (iGAS) disease within 60 days after onset of scarlet fever. Reports of scarlet fever in England during 2011–2016 were matched by residential address to persons with laboratory-confirmed iGAS infections. We identified 11 iGAS cases in ≈189,684 household contacts and a 60-day incidence rate of 35.3 cases/100,000 person-years, which was 12.2-fold higher than the background rate (2.89). Infants and contacts >75 years of age were at highest risk. Three cases were fatal; sepsis and cellulitis were the most common manifestations. Typing for 6 iGAS cases identified *emm* 1.0 (n = 4), *emm* 4.0 (n = 1), and *emm* 12.0 (n = 1). Although absolute risk in household contacts was low, clinicians assessing household contacts should be aware of the risk to expedite diagnosis and initiate life-saving treatment.

During 2014, England and Wales had a sharp increase in the incidence of scarlet fever, which by 2016 had reached 33.2 cases/100,000 person-years, the highest rate in almost 50 years (*1**,**2*). An increase in disease incidence was similarly reported from 2009 onward in Vietnam, Singapore, Hong Kong, and mainland China but has not been reported elsewhere in Europe (*1**,**3**–**7*). The cause of this increase is unknown (*1**,**5**,**8*).

Scarlet fever was once a common cause of childhood death before incidence and deaths decreased dramatically during the 19th century (*1**,**9*). Although now typically a mild disease, scarlet fever remains statutorily reportable in England to enable prediction of periods of increased incidence of invasive group A *Streptococcus* (iGAS) infection given the temporal correlation between these 2 conditions (*1**,**2*). Genomic assessment of *Streptococcus pyogenes* has furthermore demonstrated that the same strains cause scarlet fever and iGAS infection (*10**,**11*). iGAS is statutorily reportable to make contact tracing easier, given the increased risk for secondary iGAS infection among household contacts (*4**,**12**,**13*).

This study was initiated as part of a coordinated public health response to determine the cause and effect of the increase in scarlet fever in the United Kingdom (*1**,**2**,**11*). We investigated whether there is an excess risk for secondary iGAS infection in households in which a person was given a diagnosis of scarlet fever to determine whether further public health actions are required to protect contacts.

## Methods

### Study Design, Population, and Definitions

We conducted a retrospective cohort study to compare the incidence of iGAS infection among household contacts of persons with scarlet fever with the background incidence of iGAS infection in England. The cohort comprised all scarlet fever case-patients resident in England who had disease onset during January 1, 2011–December 31, 2016. Suspected cases of scarlet fever are reported by clinicians on the basis of clinical signs consistent with the condition, with or without laboratory confirmation of GAS infection. iGAS infection was defined by isolation of GAS from a normally sterile site (including blood, joint aspirates, cerebrospinal/pericardial/peritoneal/pleural fluids, deep tissue or abscess at surgery or necropsy, and bone).

A scarlet fever–iGAS household cluster was defined as a household in which a person of any age received a diagnosis of scarlet fever and, within the next 60 days, a different member of the same household received a diagnosis of iGAS infection. Case-patients resident in institutional settings were excluded. A 60-day interval was selected on the basis of preliminary analysis of the interval between onset of scarlet fever and iGAS specimen date in address-matched pairs.

### Data Sources

Demographic details of scarlet fever reports were obtained from the Public Health England (PHE) HP Zone (InFact UK, Ltd., http://hpzoneinfo.in-fact.com), a tool used nationally by health protection teams to assist case and incident management. Reports of iGAS infection were extracted from the PHE national laboratory surveillance database (Second Generation Surveillance System, https://sgss.phe.org.uk). Both datasets were sent to a National Health Service demographic batch tracing service to complete missing postcodes (typically corresponding to 15 addresses [*14*]), addresses, and patient identifiers. We sought missing postcodes for iGAS cases from the national reference laboratory database. Preliminary analysis indicated that all scarlet fever case-patients within address-matched pairs were <10 years of age. Therefore, data for the number of households in England with >1 child <10 years of age and the number of persons by single year of age in these households were provided by the Office for National Statistics Labour Force Survey (*15*) for use as denominators in risk calculations. Midyear resident population estimates for 2011 through 2016 were also obtained from the Office for National Statistics.

We obtained clinical information for cases within scarlet fever–iGAS pairs from HP Zone. National laboratory surveillance and reference laboratory data were used to identify co-infection and *emm* typing. We obtained the index of multiple deprivation decile score for each household on the basis of residential postcode (*16*). This index is a measure of relative socioeconomic deprivation based on 7 domains and provides a ranking at granular geographic level (≈1,500 residents) from the most to least deprived areas in England (*17*).

### Data Analysis

#### Identification of Household Clusters

We cleaned and analyzed data by using R version 3.2.2 (https://cran.r-project.org/bin/windows/base/old/3.2.2). Records without a postcode were excluded. We matched scarlet fever cases to all iGAS reports with a specimen date during November 1, 2010–March 1, 2017, by residential postcode. This matching enabled capture of linked iGAS cases occurring within 2 months of the first and last scarlet fever case. Cases within postcode-matched pairs without full address were removed. Addresses of remaining matched pairs with an interval between onset of scarlet fever and iGAS specimen date <60 days were visually scrutinized to exclude institutional settings and confirm co-location.

The iGAS and scarlet fever datasets were deduplicated after matching to ensure that all sequential specimens were considered in identifying temporal links between cases. An interval >14 days between specimen dates was considered a new episode for iGAS and >30 days between onset dates for scarlet fever. To supplement household clusters identified from address matching, we reviewed GAS clusters and outbreaks recorded on HP Zone and the reference laboratory outbreak database over the same period.

### Calculation of Risk

We estimated the average number of household contacts of scarlet fever cases by dividing the total number of persons living in households with a child <10 years of age in England during the study period by the number of households and subtracting 1 to account for the case-patient being a household member. We multiplied this figure by the number of scarlet fever cases to estimate the total number of contacts and calculated the person-years at risk over 60 days. We calculated the incidence of iGAS infection among scarlet fever household contacts by dividing the number of iGAS cases among these contacts by the number of person-years at risk and used Poisson distribution to define 95% CIs. The background rate of iGAS infection was based on the total number of iGAS cases in England. We repeated this analysis by year and age group. We conducted a sensitivity analysis to investigate the effect of increasing the average household size by up to 3 household members (3.8/household to 6.8/household).

### Ethics Considerations

Ethics approval was not required because we analyzed only routinely collected data. PHE has the authority to collect and process confidential patient information for communicable disease surveillance and control under Section 251 of the National Health Service Act of 2006.

## Results

**Figure 1:**
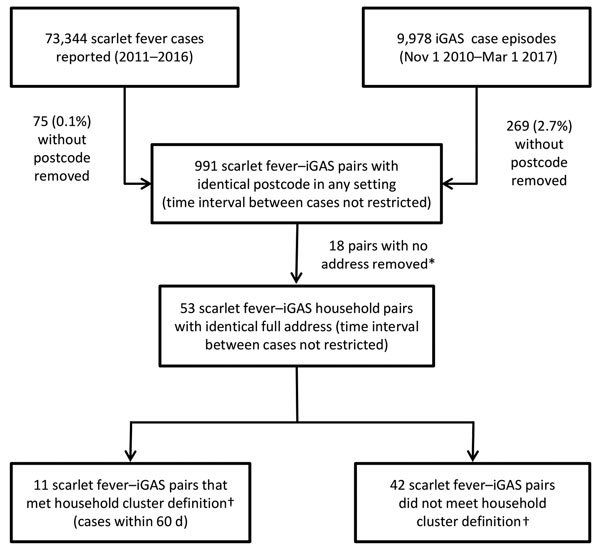
Summary of records included at each stage of the matching process of scarlet fever and iGAS cases, England 2011–2016. *Interval between excluded pairs was >60 days. †A household cluster was defined on the basis of a person being given a diagnosis of scarlet fever and a different member of the same household given a diagnosis of iGAS infection for which onset of iGAS symptoms occurred within 60 days after onset of scarlet fever. iGAS, invasive group A *Streptococcus* infection.

A total of 73,344 scarlet fever cases were reported to PHE during 2011–2016. Of the 9,978 episodes of iGAS infection extracted for address matching to scarlet fever cases, 2.7% (269) were excluded due to a missing address; a higher proportion of cases before 2014 had a missing postcode than cases from 2014 onward (4.4% vs. 1.4%, χ^2^ 85.2, df 1, 95% CI 2.3–3.7; p<0.0001). We identified 991 scarlet fever–iGAS pairs with identical postcodes in any setting (including institutions); 1.8% (18) did not have a full address and were excluded from further analysis (onset interval range 93–1,893 days) (Figure 1). Of the remaining 973 pairs, 53 were resident in a private home and confirmed as being at the same address; iGAS cases occurred after scarlet fever onset for 28 of 53 pairs.

**Figure 2:**
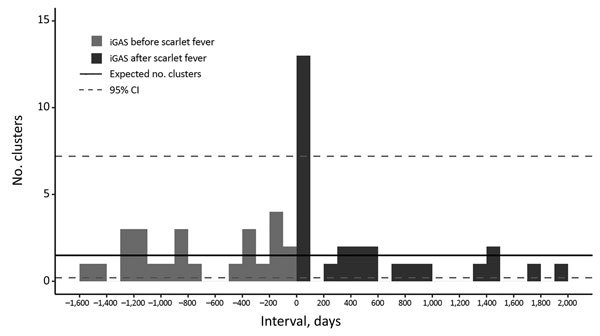
Distribution of time interval between onset of scarlet fever and iGAS within address-matched pairs (n = 53) and expected number of clusters, England 2011–2016. Exploratory analysis was used to identify the period of excess numbers of iGAS cases before review of case records; iGAS cases might be linked to >1 scarlet fever case episode in the same household. The background iGAS rate was 2.88 cases/100,000 person-years; 95% CI is based on 2 expected cases/100 days. There were 189,684 scarlet fever household contacts. iGAS, invasive group A *Streptococcus* infection.

A pronounced increase in the number of pairs was evident within the first 100 days after onset of scarlet fever (Figure 2): 13 pairs identified, compared with an expected 1.5 (95% CI 0.2–7.2) iGAS cases based on background iGAS infection rates. All 13 pairs were within 60 days, and on review of case details, 11 met the household cluster definition. No clusters were identified through review of the national case management system or the reference laboratory database. Two of the 25 pairs in which iGAS occurred before scarlet fever had an interval between cases <60 days, a rate of 6.4 iGAS cases/100,000 person-years and twice the background rate (rate ratio [RR] 2.2, 95% CI 0.6–8.9). Fifty-one pairs with onset dates within 60 days had the same postcode but were resident in different private homes.

We identified 18 persons given a diagnosis of iGAS and scarlet fever. The median interval between onset of scarlet fever and iGAS specimen date was 155 days (range 5–1,488 days). Sixteen cases had an interval >100 days, and 7 cases had iGAS infection after a diagnosis of scarlet fever.

### Characteristics of Household Clusters

**Figure 3:**
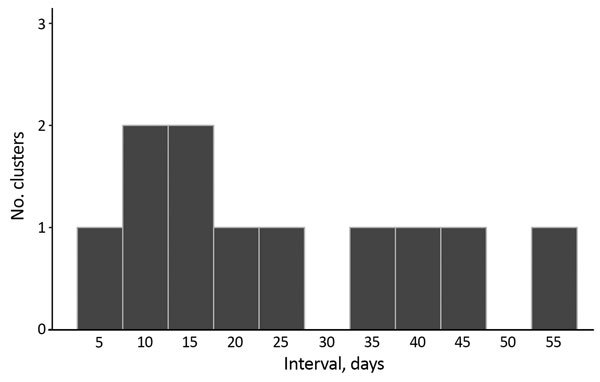
Distribution of time intervals between onset of scarlet fever and invasive group A *Streptococcus* infection within each pair meeting the household cluster definition (n = 11), England 2011–2016.

All household clusters were composed of 1 scarlet fever case and 1 iGAS case and occurred after March 2014. The median interval between onset of scarlet fever and of iGAS infection was 18 days (range 3–54 days) (Figure 3). Five iGAS cases occurred in parents of children with scarlet fever and 4 in siblings; the relationship to the scarlet fever case-patient was not recorded for 2 clusters (iGAS case-patients 86 and 26 years of age) (Table 1). Five iGAS case-patients had sepsis and 3 had cellulitis. Five of the 11 iGAS case-patients had an underlying chronic condition, and 1 had an acute infection with influenza. Three of the iGAS case-patients died; 2 of them had predisposing conditions (arthritis, diabetes, and atypical mycobacterial infection). The median household size was 4 persons (range 4–6 persons). Seven of 11 households were in the 30% most deprived neighborhoods in England, and 3 were in the 30% least deprived.

**Table 1:** Characteristics of 11 iGAS case-patients within household clusters, England, 2011–2016*

Characteristic	No. case-patients		
Total	Male sex	Female sex
Age, y			
<1	2	1	1
1–18	2	0	2
19–50	6	3	3
>75	1	1	0
Total	11	5	6
Relationship to scarlet fever case-patient			
Parent	5	3	2
Sibling	4	1	3
Unknown	2	1	1
Acute health conditions at time of diagnosis			
Influenza A	1	NR	NR
Chronic health condition at time of diagnosis			
Arthritis	1	NR	NR
Crohn’s disease	1	NR	NR
Premature birth	1	NR	NR
Diabetes	1	NR	NR
Atypical mycobacterial infection	1	NR	NR
Asplenia	1	NR	NR
Multiple unnamed conditions	1	NR	NR
No concurrent conditions	6	NR	NR
Died	3	NR	NR
Clinical manifestation			
Sepsis	5	NR	NR
Cellulitis	3	NR	NR
Septic arthritis	1	NR	NR
Other invasive infection (unspecified)	2	NR	NR
iGAS *emm* typing			
1.0	4	NR	NR
4.0	1	NR	NR
12.0	1	NR	NR
Untyped	6	NR	NR

*iGAS, invasive group A *Streptococcus* infection; NR, not reported.

Strain typing was available for 6 of the iGAS household cluster cases, 4 of which were *emm* 1.0 and 1 each were *emm* 4.0 and *emm* 12.0. Typing results for scarlet fever isolates were not available.

### Calculation of Risk

All scarlet fever case-patients within clusters were <10 years of age. Therefore, we restricted analysis of risk for iGAS infection to household contacts of scarlet fever case-patients <10 years of age (n = 66,191). We estimated that these case-patients had 189,684 household contacts (average household size 3.9 persons). We estimated the incidence of iGAS infection among these contacts to be 35.3 cases/100,000 person-years (95% CI 17.6–63.2 cases/100,000 person-years) compared with a background incidence of iGAS in England (all ages) of 2.9 cases/100,000 person-years (Table 2). Therefore, the rate of iGAS infection in household contacts of persons with scarlet fever was 12 times higher than the background rate in England over the same period (RR 12.2, 95% CI 6.7–22.1) (Table 2). The highest absolute rates were for infants (138 cases/100,000 person-years, 95% CI 16.7–496.8 cases/100,000 person-years) and persons >75 years of age (1,419 cases/100,000 person-years, 95% CI 35.9–7907.3 cases/100,000 person-years), although these rates were based on a small number of cases (Table 3). RR was highest for contacts 11–17 years of age (RR 43.9, 95% CI 6.1–313.7) and contacts >75 years of age (RR 139.2, 95% CI 19.6–988.5) (Table 3).

**Table 2:** Risk for iGAS infection among household contacts of scarlet fever case-patients <10 years of age by year compared with background iGAS incidence, England, 2011–2016*

Year	No. scarlet fever cases	Estimated no. contacts	No. iGAS cases in contacts†	Attack rate/100,000 person-years (95% CI)	Background iGAS incidence/100,000 person-years	Rate ratio (95% CI)
2011	3,128	8,929	0	0.0 (0.0–251.5)	2.29	NA
2012	4,632	13,073	0	0.0 (0.0–171.8)	2.35	NA
2013	5,204	14,778	0	0.0 (0.0–152.0)	2.99	NA
2014	16,394	47,015	3	38.8 (8.0–113.5)	2.29	16.9 (5.5–52.6)
2015	18,022	51,454	3	35.5 (7.3–103.7)	3.48	10.2 (3.3–31.7)
2016	18,811	54,435	5	55.9 (18.2–130.5)	3.91	14.3 (5.9–34.7)
Total	66,191	189,684	11	35.3 (17.6–63.2)	2.89	12.2 (6.7–22.1)

*iGAS, invasive group A *Streptococcus* infection; NA, not applicable. †During the 60 days after onset of scarlet fever in the household.

**Table 3:** Risk for iGAS among household contacts of 66,191 scarlet fever case-patients <10 years of age compared with background iGAS incidence by age, England, 2011–2016*

Age of contacts, y	Estimated no. contacts	No. iGAS cases in contacts†	Attack rate/100,000 person-years (95% CI)	Background iGAS incidence/100,000 person-years	Rate ratio (95% CI)
<1	8,853	2	137.5 (16.7–496.8)	6.42	21.4 (5.31–86.1)
1–10	28,660	1	21.2 (0.5–118.3)	2.84	7.5 (1.1–53.1)
11–17	22,209	1	27.4 (0.7–152.7)	0.58	43.9 (6.1–313.7)
18–50	122,801	6	29.7 (10.9–64.7)	1.69	18.4 (8.4–41.1)
51–74	6,733	0	0 (0–333.5)	3.59	0
>75	429	1	1,419.2 (35.9–7.907.3)	10.20	139.2 (19.6–988.5)

iGAS, invasive group A *Streptococcus* infection. †During the 60 days after onset of scarlet fever in the household.

Sensitivity analysis, increasing the average household size from 3.8 to 6.8 members, reduced the RR for iGAS in scarlet fever household contacts relative to the background iGAS rate to 6 (95% CI 3.3–10.8). The rate of iGAS infection in scarlet fever household contacts before the period of increased scarlet fever incidence (2011–2013), 0 cases/100,000 person-years (95% CI 0.0–61.1), was not significantly different for the period of increased incidence (2014–2016), 43.7 cases/100,000 person-years (95% CI 21.8–78.4).

## Discussion

Our study identified a low risk for iGAS infection among household contacts of scarlet fever cases (35.3 cases/100,000 person-years). However, this risk was increased when compared with the background risk. Eleven iGAS cases occurred among ≈189,684 contacts during the 60 days after scarlet fever onset; 1.5 cases would have been expected on the basis of a background rate of 2.9 cases/100,000 person-years. Small numbers of cases preclude robust subgroup analysis but excess risk was highest in contacts >75 and 11–17 years of age. Although the absolute risk was low, the effect of these infections was severe; 3 deaths were reported. These findings have implications for other countries reporting a high incidence of scarlet fever.

Half the secondary iGAS cases occurred in parents and one third occurred in siblings of scarlet fever case-patients. A previous study showed a slight excess in scarlet fever incidence in young adult women, possibly explained by caring responsibilities for children with the infection (*1*). We did not observe a similar pattern for iGAS; 3 of 5 cases in parents were in men. Contacts >75 years of age had the highest absolute risk for development of iGAS. Although the background rate for iGAS was highest in elderly persons, because there were only an estimated 429 household contacts of scarlet fever case-patients within this age group, 1 secondary iGAS case in this group translated into a high attack rate.

An estimated 5 million grandparents have regular childcare responsibilities in the United Kingdom (*18*). We were unable to assess the risk for grandparents not living in the same household and are likely missing a major group potentially at risk through contact with scarlet fever case-patients. Our identification of 51 postcode-matched pairs with different addresses suggests a possible increased risk for iGAS infection in the neighborhood of scarlet fever case-patients and warrants further assessment. Although a proportion of the observed secondary household iGAS risk might be caused by transmission in wider social networks, the fact that parents constituted most secondary iGAS cases suggests that transmission within the home underpins these clusters because parents are less likely than children to be exposed to scarlet fever outside the home.

Almost half the iGAS case-patients reported underlying chronic conditions, although diabetes, Crohn’s disease, and arthritis are common, which limits the potential to target public health actions. A broad range of clinical initial manifestations were reported for iGAS infections within clusters, including skin and soft tissue and joint infections. Overcrowding is a known predisposing factor for *S. pyogenes* infections (*4*), but we did not find evidence of this factor in clusters who lived in average-sized households (median 4 occupants), although this information was not available in public health records for 4 of 11 households.

Pharyngeal carriage of GAS among close contacts of persons with invasive infections has been demonstrated (*19*); person-to-person transmission of GAS occurs by respiratory droplets or skin contact (*4*). We do not assume that iGAS infection was necessarily acquired from the child with scarlet fever. Other members of the household (symptomatic or asymptomatic) might have been the source to either case-patient, particularly given the long period of risk (60 days) and that the scarlet fever case-patient probably received treatment.

We examined the risk for secondary iGAS infection before and after onset of scarlet fever, without preconceptions as to the length of identified period of excess risk. Transmission of GAS within the household for 60 days is plausible; back-and-forth transmission between household members is well-described (*20**–**22*) and has been demonstrated to occur over a 10-month period (*21*). Transmission from an asymptomatic carrier can occur up to several weeks after acquisition although communicability is lower than from symptomatic cases (*19**,**23*). An ongoing study in London aims to assess GAS carriage in family members of scarlet fever case-patients (*24*).

Environmental reservoirs have been implicated in hospital (*25**,**26*), nursery (*27*), and care home outbreaks (*4**,**28*), but the duration of viability in the environment is unknown. Survival on dry surfaces has been demonstrated after several months; therefore, public health messaging should include advice on infection control in households, particularly where there are susceptible persons (*29**,**30*).

Although it was not the focus of this study, we observed a slight excess risk for iGAS occurring before scarlet fever. Public health guidelines on the management of iGAS infection in the United Kingdom recommend that household contacts are advised to visit their general practitioner (GP) for assessment if they have symptoms of GAS infection in the 30 days after onset in the index case-patient. Therefore, these scarlet fever case-patients should have already been under surveillance, potentially increasing the likelihood of their diagnosis.

The number of contacts of scarlet fever case-patients was the main source of uncertainty in our risk estimation. If households that have scarlet fever case-patients differ in size from the average household with children, we could have underestimated or overestimated the risk. However, the risk for iGAS infection would still have been 6 times higher than background if the average household size was increased to 7 members. Coupled with our observation that household clusters had a median size of 4 (compared with 3.9 for all households in England), it is unlikely the uncertainty about numbers of contacts could account for the increase in risk observed.

Failure to match iGAS to scarlet fever cases could have occurred because of missing postcodes (3% of iGAS cases), errors in the postcode or address, or because the traced postcode represents the current address and might be different from that at the time of infection. Our finding that all clusters occurred after the increase in scarlet fever during 2014 was possibly influenced by this factor, given that postcode completion was higher in the later years of the study and enabled identification of clusters. We did not adjust for residence in a long-term care facility or hospitalization in the background risk calculation: 3.5% of iGAS cases in England (2009–2010) were estimated to be acquired in long-term care facilities, and 6% of these infections were estimated to be acquired in hospitals. Residents of long-term care facilities had a 6-fold higher risk for iGAS infection than community residents (*31**,**32*). Including institutionally acquired infections slightly increased the background iGAS risk in this study. 

We used clinical reports of scarlet fever and recognized that a proportion of reported cases might have had other infections, which has the potential to influence the risk estimate in either direction. Although only ≈50% of scarlet fever consultations in primary care in England are formally reported (*1*), this finding would not influence the risk estimate because it was based on iGAS cases that occurred in contacts of only the reported cohort. We did not capture the burden of disease associated with severe noninvasive GAS infections (GAS isolated from a nonsterile site), although these infections are estimated to comprise only 1% of all severe GAS infections (*33**,**34*).

We assessed the risk for secondary iGAS infection for household contacts to be low. However, potential severity is high. The prodrome for iGAS infection can be nonspecific, and the disease can progress rapidly; therefore, increasing the index of suspicion in specific circumstances or groups at increased risk might expedite diagnosis and commencement of life-saving treatment (*4**,**35*). Offering antimicrobial drug prophylaxis to household contacts to eradicate carriage and treat incipient infection could reduce the risk for iGAS infection. However, the unintended consequences of large-scale increased use of antimicrobial drugs, heightened patient anxiety, the effect on GP workload, and the lack of evidence on effectiveness make this option disproportionate given the low overall risk estimated. Antimicrobial drug prophylaxis could be targeted to high-risk contacts, such as elderly persons and infants. However, there is considerable uncertainty for the risk estimate for these groups because of the small number of secondary iGAS cases. Providing information on signs and symptoms of iGAS infection to patients or parents at the point of scarlet fever diagnosis to accelerate self-referral for medical assessment could be effective but has the potential to increase anxiety for many persons and increase presentations of worried healthy persons to GPs and emergency departments at scale. Increasing awareness among frontline clinicians assessing patients of this increased risk to improve early identification and treatment of cases is perhaps the most proportionate and feasible response on the basis of available data. Information could also be made available to the public through patient-facing websites provided that messages are worded carefully so as not to increase anxiety.

We recommend repeating this analysis at regular intervals to monitor and increase precision around our estimated risk. Enhanced surveillance of iGAS patients should include questions on the number of contacts and recent scarlet fever infections in the household. This information would help address some of the methodological uncertainties around the number of contacts and enable assessment of the attributable risk in the context of other risk factors. Of ≈10,000 iGAS cases identified during our study, only 11 were associated with scarlet fever contact: as such, a proportionate response to further investigations is warranted. Although increases in iGAS infection have been observed during the latter period of the scarlet fever upsurge (2016 onward), these increases follow a longer-term trend of increasing iGAS infection in England, and the connection with increased scarlet fever activity remains unclear (*36*).

It is likely that contact with other superficial manifestation of GAS infection would also increase the risk for iGAS infection. However, the mixed etiology for these conditions and lack of microbiological testing make this potential risk difficult to assess. Nonetheless, with drives to reduce antimicrobial drug treatment for conditions such as pharyngitis to relieve selection pressure favoring antimicrobial drug resistance, understanding the possible repercussions for the patient and wider community are essential.

In conclusion, we identified an excess risk for iGAS among household contacts of scarlet fever case-patients, although we assessed the overall risk to be low. We recommend that frontline clinicians maintain heightened awareness of the risk for iGAS in scarlet fever contacts when assessing patients. Further research to tighten our risk estimates and improve our understanding of transmission patterns in households will inform future prevention strategies.
